# Epitope mapping of the protease resistant products of RT-QuIC does not allow the discrimination of sCJD subtypes

**DOI:** 10.1371/journal.pone.0218509

**Published:** 2019-06-17

**Authors:** Gabriele Piconi, Alexander H. Peden, Marcelo A. Barria, Alison J. E. Green

**Affiliations:** The National CJD Research & Surveillance Unit, Centre for Clinical Brain Sciences, University of Edinburgh, Edinburgh, Scotland, United Kingdom; Deutsches Zentrum fur Neurodegenerative Erkrankungen, GERMANY

## Abstract

Sporadic Creutzfeldt-Jakob disease (sCJD) is a transmissible, rapidly progressive and fatal neurodegenerative disease. The transmissible agent linked to sCJD is composed of the misfolded form of the host-encoded prion protein. The combination of histopathological and biochemical analyses has allowed the identification and sub-classification of six sCJD subtypes. This classification depends on the polymorphic variability of codon 129 of the prion protein gene and the PrP^res^ isotype, and appears to be associated with neuropathological and clinical features. Currently, sCJD subtyping is only fully achievable *post mortem*. However, a rapid and non-invasive method for discriminating sCJD subtypes *in vita* would be invaluable for the clinical management of affected individuals, and for the selection of participants for clinical trials. The CSF analysis by Real Time Quaking Induced Conversion (RT-QuIC) reaction is the most sensitive and specific *ante mortem* sCJD diagnostic test available to date, and it is used by a number of laboratories internationally. RT-QuIC takes advantage of the natural replication mechanisms of prions by template-induced misfolding, employing recombinant prion protein as reaction substrate. We asked whether epitope mapping, of the RT-QuIC reaction products obtained from seeding RT-QuIC with brain and CSF samples from each of the six molecular subtypes of sCJD could be employed to distinguish them and therefore achieve *in vita* sCJD molecular subtyping. We found that it is possible to distinguish the RT-QuIC products generated by sCJD biological samples from the ones generated by spontaneous conversion in the negative controls, but that different sCJD subtypes generate very similar, if not identical RT-QuIC reaction products. We concluded that whilst RT-QuIC has demonstrable diagnostic value it has limited prognostic value at this point in time.

## Introduction

Prions are the infectious agents associated with Transmissible Spongiform Encephalopathies (TSEs) or prion diseases. Prion diseases are transmissible, rapidly progressive and invariably fatal neurodegenerative diseases affecting humans and other mammals. According to the protein only hypothesis [1,2], prions are constituted by the misfolded form of the host prion protein (PrP^C^), a copper binding glycoprotein bound by a glycophosphastidyl-inositol (GPI) anchor to the outer leaflet of cell membranes, which is constitutively expressed in the central nervous system (CNS).

The misfolded PrP scrapie (PrP^Sc^) isoforms, replicates inside a host by imposing their conformation on PrP^C^. The conformational change of PrP^C^ to PrP^Sc^ makes the latter insoluble in non-denaturing detergents and partially resistant to proteases such as protease K (PK), a broad-spectrum serine and threonine protease. PK is routinely used in prion research because the product of the digestion of PrP^Sc^ with PK, referred to as resistant PrP (PrP^res^), is used for the molecular characterisation of prion diseases [3].

In humans, the most common form of prion disease is sporadic Creutzfeldt-Jakob disease (sCJD) accounting for 85%-90% of all human prion disease cases. The remaining 10–15% of human prion disease cases are mainly due to genetic forms, linked to point or insertional mutations in the prion protein gene *PRNP*. Although of high concern to public health, acquired forms, due to inadvertent human-to-human transmission or zoonotic transmission, are the rarest [4,5].The clinical presentation and disease course of sCJD, broadly correlate with six molecular subtypes usually referred to as sCJD MM1/MV1, MM2c (c for cortical), MM2t (t for thalamic), MV2, VV1, and VV2. Currently, the biochemical identification of sCJD subtypes is achieved *post mortem*, as it requires the analysis of brain tissue samples. These sCJD biochemical subtypes are defined by the genotype at *PRNP* polymorphic codon 129 (encoding methionine [M] or valine [V]) in combination with the molecular weight of the unglycosylated PrP^res^ found in brain tissue samples and scored by western blot (WB) with an anti-PrP monoclonal antibody [6,7] (Type 1 = 19 kDa, Type 2 = 21 kDa). Animal studies have shown that these six sCJD subtypes comprise four major human strains of prions (M1, M2, V1, V2) as defined by the length of incubation time, susceptibility and disease phenotype in selected inbred strains of mice as well as the conservation of characteristic neuropathological lesion patterns upon serial passage in the same host [8,9].

To explain the phenotypic characteristic of different strains, it has been proposed that these are enciphered within a subset of all the possible PrP^Sc^ conformations and their interaction with the cellular milieu [10]. A corollary to this hypothesis, is that the two PrP^res^ types, the discrimination of which contribute to sCJD biochemical subtyping, might arise from different PrP^Sc^ conformations that in turn dictate which sites on the structure of PrP^Sc^ are available to PK activity.

Cell-free conversion (CFC) systems are methodologies that emulate prion replication *in vitro*. CFCs were originally developed to test the protein only hypothesis [11] and investigate prion biology [12,13] and have subsequently found application in clinical practice as an aid to prion disease diagnosis. The Real Time Quaking Induced Conversion reaction (RT-QuIC) is a 96-well plate-based CFC system. It is characterised by the use of bacterially expressed and purified recombinant prion protein as reaction substrate. Heat and intermittent shaking are the energy inputs and the registered output signal consists of fluorescence readings over time. The fluorescent signal output is provided by the amyloid-specific dye Thioflavin T (ThT), which is present in the reaction mixture. When a prion containing sample (termed seed) is present, it induces the recombinant substrate to alter its conformation and form ThT binding-competent aggregates. Ultimately, RT-QuIC signal output is an indirect observation of the kinetics of the prion induced misfolding and aggregation of the recombinant substrate.

Since its first introduction, RT-QuIC has been applied to human prion disease diagnosis [14–16]. To maximize RT-QuIC diagnostic specificity (i.e. minimize false positive), efforts have been put into optimising the RT-QuIC reaction conditions to increase the kinetic difference between sCJD and non-CJD seeded reactions as well as to avoid the spontaneous aggregation of the recombinant substrate in unseeded reactions.These efforts resulted in the optimisation and international harmonisation of an *ante mortem* sCJD diagnostic RT-QuIC protocol, based on cerebrospinal fluid (CSF) analysis [17,18].

A widely used and characterised substrate for the sCJD RT-QuIC diagnostic protocol is the full-length hamster prion protein (a.a. 23–231) [19]. However, by adapting the reaction conditions, recombinant prion protein from other mammals have been used, highlighting the flexibility of RT-QuIC in its ability to detect human and non-human prions from different sources [20][21].

RT-QuIC has shown to be the most sensitive and specific *ante mortem* diagnostic test for sCJD available at this point in time, prompting researcher to ask whether it might be employed to discriminate sCJD subtypes. The discrimination of the sCJD subtypes by RT-QuIC would provide an outlook on the disease progressionas well as diagnostic information, which would inform the clinical management of affected individuals. Moreover, sCJD molecular subtypes ascertained *in vita* could be used as inclusion criteria for the recruitment of patients into future clinical trials.

Testing a limited number of sCJD brain samples, Peden and colleagues [22], firstly reported a lower RT-QuIC seeding potency for the rarer sCJD subtypes MM2 and VV1 when compared with the more common MM1 / MV1 subtype. In subsequent studies, however, the stratification of sCJD diagnostic RT-QuIC results (i.e. the RT-QuIC analysis of sCJD CSF samples) obtained from multiple laboratories, by either codon 129 genetic status, or sCJD subtype, showed little or no differences in terms of specificity and sensitivity [23–25]. In contrast, a recent adaptation of RT-QuIC, employing an N-terminally truncated form of the Hamster recombinant PrP (i.e. 90-231Ha-rPrP) as a reaction substrate, revealed sCJD subtype-specific kinetics of aggregation, when reactions were seeded with CSF samples [26]. Adding to this, there is evidence that the misfolded form(s) of recombinant PrP generated during the RT-QuIC reaction (herein referred to as RT-QuIC reaction products) can retain structural features of the prion seed that generated them allowing for their *in vitro* identification [27–29]. These findings suggest that sCJD subtypes may be discriminated by RT-QuIC based on the biochemical characteristics of the generated aggregates, however it is not clear at this point in time whether the commonly employed routine CSF analysis by RT-QuIC can be extended to subtype sCJD using the same criteria used for post-mortem PrP^res^ subtyping from brain tissue. Or to put the same point in more theoretical terms: whether conformational and perhaps even strain characteristics are conserved during quaking induced conversion.

By seeding the diagnostic RT-QuIC reaction with brain and CSF samples accounting for the most common sCJD subtypes (MM1/MV1, MM2, MV2, VV1, VV2), we have analysed the RT-QuIC reaction products for their resistance to limited proteolysis and investigated the length of the resistant fragments via epitope mapping using a set of six commercially available antibodies, including those usually employed for sCJD molecular typing.

## Materials and methods

### Biological samples and sample inclusion criteria

All the human biological samples were provided by the National CJD Research and Surveillance Unit (NCJDRSU) Brain and Tissue Bank in Edinburgh, UK (part of the MRC Edinburgh Brain Bank) ethical approval (Scotland A REC 05/MRE00/67, Edinburgh Brain Bank 16-ES-0084). The inclusion criteria for the sCJD and non-CJD samples used in this study were based on of the appropriate consent for research for each individual case and the availability of frozen tissue. The cases selected comprised three frozen brains each of sCJD subtypes MM1, MM2, MV1, MV2, VV2 and two of subtype VV1 (using the Parchi subtypes nomenclature; 6). In addition, one case reported to the NCJDRSU with neurological symptoms but no signs of prion disease (non-CJD) following pathological investigation, was selected as negative control. Small pieces of frontal cerebral cortex were sampled from all the above-mentioned cases.

The collected tissue samples were homogenised to 10% weight/volume in Phosphate Buffered Saline (PBS) supplemented with 150 mM NaCl, 1 mM ethylene-diamine-tetra-acetic acid (EDTA), 0.5% Triton X-100 and Complete Protease Inhibitor (Roche) using a FastPrep-24 automated homogeniser (MPbio) and lysing matrix D.

The levels of PrP^res^ in each 10% brain homogenate (10% BH) was evaluated by densitometric analysis following Western blot (WB) analysis using purified recombinant PrP as a standard. The amounts of brain used to seed individual RT-QuIC reactions were normalised to 100 fg of PrP^res^. All the 10%BH were diluted in PBS, the range of 10%BH dilution factors used after normalisation was from 10^−3.8^ to 10^-5.7^ and the mean of this dilution factor range was adopted as the dilution factor for the non-CJD 10%BH used as negative control. PBS was used in the unseeded samples as a control for the spontaneous aggregation of the substrate.

CSF samples were selected on the basis of volume availability, testing positive in a previous RT-QuIC analysis and being from patients with neuropathologically-confirmed sCJD with a known sCJD subtype. The selected CSF sample set included three examples of sCJD MM1, MV2 and VV2 and two examples of MV1 and MM2 (cortical rather than thalamic variant). Two additional CSF samples were selected as negative controls based on being assigned with diagnosis of a non-prion related neurological disease, sample volume availability and testing negative in a previous RT-QuIC analysis. A volume of 15 μL of neat CSF were used to seed individual reaction wells [17].

### Recombinant full-length hamster prion protein purification

An individual batch of full-length Syrian Golden Hamster recombinant prion protein (FLHa-PrP, residues 23–231) was purified and used in this study. Purification protocol was as previously described [11], in brief: Syrian Golden Hamster *PRNP* (accession K02234) expression and translation from transformed *Escherichia coli* Rosetta cells (Invitrogen), was obtained with Overnight Express Instant TB medium (Merck Novagen). Bacteria were collected and inclusion bodies (IB) isolated in pellets by means of Bug Buster Master Mix (Merck Novagen). IB pellets were resuspended and bound to Ni-NTA Superflow resin beads (Qiagen) in denaturing conditions. Protein was refolded on the column and subsequently eluted. Concentration was adjusted by means of Amicon Ultra-15 centrifugal Filter Units (Merck Millipore) and one millilitre aliquots were prepared, flash frozen and stored at -80°C until use.

### RT-QuIC reaction and collection of RT-QuIC reaction products

The RT-QuIC reaction was performed in a sealed 96 well plate with clear bottom (Thermo-Fisher) incubated at 42°C for 90 h in a FLUOstar Optima (BMG Labtech) plate reader. During the incubation, the plate reader performed cycles of intermittent shaking (1 minute shake, 1 minute rest) and took fluorescence readings from the bottom of each well every 15 min (450 nm excitation, 480 nm emission, gain 2000) [17].

All the RT-QuIC reaction buffer components were purchased as concentrated stock solutions from Sigma-Aldrich and diluted in cell culture grade water. RT-QuIC reaction buffer composition was as follows: PBS (5 mM phosphate, 154 mM NaCl), 170 mM NaCl, 1 mM EDTA and 10 μM ThT. Because PBS contains NaCl, the final RT-QuIC reactions contained 324mM NaCl. Thioflavin T (ThT) solution was prepared monthly as a 10 mM solution which was syringe filtered through a 0.22 μm filter (Merck Millipore) and kept at +4°C in the dark. Prior to use, ThT was diluted to 1 mM and used at a final concentration of 10 μM in the RT-QuIC reactions. Recombinant FLHa-rPrP substrate was thawed (1 hour on ice followed by 30 minutes at room temperature) and filtered through a 100 kDa centrifuge filter (Nanosep, Pall). Following filtering, the concentration of the substrate was calculated from measures of absorbance at 280 nm taken on a dilution 1:2 in 0.1% sodium dodecyl sulphate in PBS (0.1%SDS/PBS) using a NanoDrop OneC (Thermo-Fisher), blanking with 0.05%SDS/PBS.

The FLHa-rPrP was then incorporated to the reaction buffer at 0.1 μg/μL, generating a bulk reaction mixture that was subdivided between reaction wells. The final reaction volume was 100 μL per well including seed. The RT-QuIC reactions were seeded with 15 μL of CSF or 2 μL of 10% BH serially diluted in PBS containing 100 fg of PrP^res^. After preparing the RT-QuIC reaction plate, a volume of PBS accounting for the absence of seed (15 μL of CSF or 2 μL of 10% BH dilution) was added to the excess of reaction mixture. These mock reaction mixtures were then stored in 1.5 mL polypropylene tubes (APEX NoStick Alpha Laboratories) at -20°C. These samples, termed “Mix”, were used as RT-QuIC untreated controls in subsequent immunochemical analyses of the RT-QuIC reaction products.

At the end of each RT-QuIC, the sealed plate was extracted from the plate reader and left to equilibrate at room temperature. Condensation was spun down by centrifugation at 2505 x g for 1 min. The content of the wells was collected by carefully scraping the well surfaces with a disposable pipette tip, prior to transferring the liquid suspension to a storage tube. A minimum of six technical replicates per seed were prepared on an individual plate. Collected samples were stored in 1.5 mL polypropylene tubes (APEX NoStick Alpha Laboratories) at -20°C until use.

### SDS PAGE and Western blot

All SDS-PAGE analyses were performed using pre-cast 10% bis-tris gels (NuPAGE). Following preparation, samples were incubated at 100°C for 10 minutes in sample loading buffer (LDS4X, NuPAGE) or in LDS4X supplemented with 2% volume/volume (v/v) 2-mercaptoethanol (LDS4XβMA) and loaded onto the gel. Electrophoretic separation was performed in MES-SDS buffer (NuPAGE) at 200V for between 35 and 45 minutes.

Gels were blotted on polyvinylidene fluoride membrane (BioRad Immun-Blot PVDF) by means of wet transfer (1 hour, 0.8 A constant) using Towbin buffer [30]. This system permitted the transfer of up to six gels onto one membrane [31]. Following transfer, PVDF membranes were blocked overnight in a 5% solution of non-fat dry milk in 0.1% Tween 20 Tris buffered saline (TBST). The following day, blocking solution was removed and at this stage, if multiple gels had been transferred onto the same membrane, the membrane was split into patches corresponding to individual gels. The membranes were incubated according to the conditions specific for the primary antibody of choice. Performing the analysis of primary antibodies on samples that had been transferred together increased the reliability of this comparison by eliminating any variation in transfer efficiency. At the end, each membrane was washed three times for 10 minutes with fresh TBST and subsequently incubated for 1 hour at RT and gentle shaking in a solution of horseradish peroxidase conjugated secondary antibody (Novex, Goat anti-Mouse IgG) 1:25000 in TBST. After one hour, the membrane was washed with two 10 minutes washes and two 5 minutes washes with TBST before applying enhanced chemiluminescence system (ECL Prime, Amersham). The signal was detected by digital imaging with ChemiDoc XRS+ (BioRad).

### Monoclonal antibodies used

**Fig 1:**
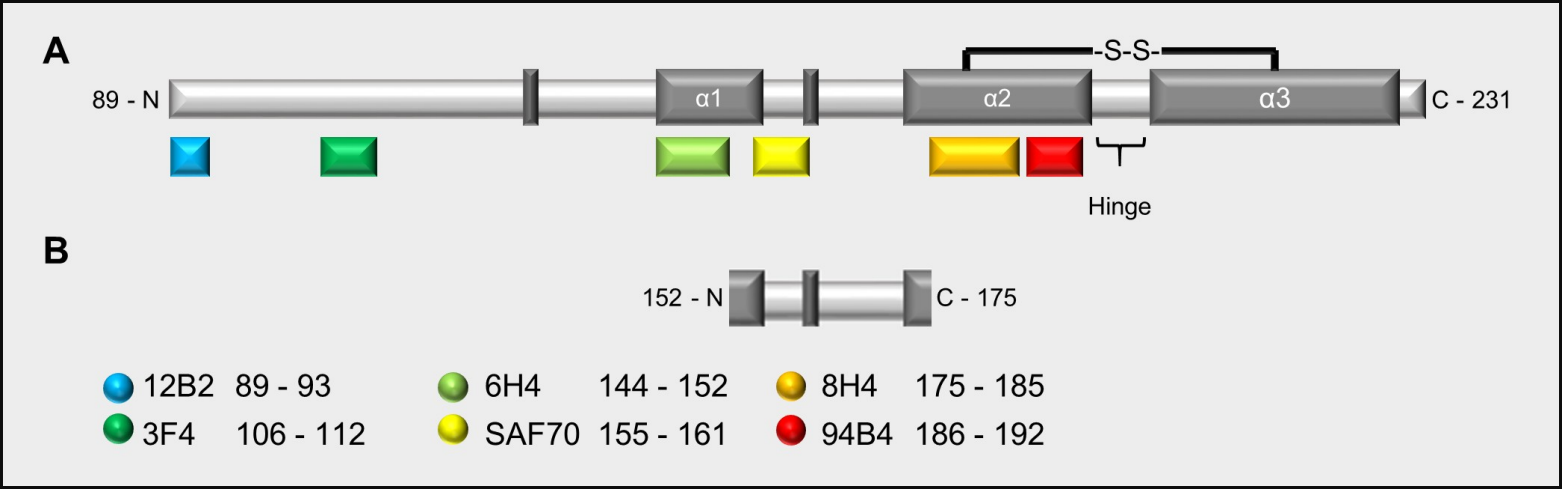
Diagram of the recombinant Hamster prion protein summarizing structural features and epitope locations. (A) diagram of the recombinant Hamster prion protein truncated at position 89. Dark grey boxes represent secondary structures. S-S indicates the disulphide bridge that connects alpha helix 2 (α2) to alpha helix 3 (α3) generating the hairpin loop. H indicates the hinge of the loop. Antibodies are listed and colour-matched with boxes indicating the location of the recognised epitope on the sequence. (B) Minimal extent of the fragment detected by SAF70 following PK digestion of sCJD seeded RT-QuIC products.

Anti-PrP monoclonal antibody (mAb) 3F4 (epitope 106–112, Hamster sequence) was purchased from Millipore as a 1 mg/mL solution and was used at a dilution of 1:10000. PrP^res^ Type 1 specific mAb 12B2 (epitope 89–93) and C-terminus specific 94B4 (epitope 186–192) were purchased from the Central Veterinary Institute at Wageningen University Netherlands, EU, as 1 mg/mL solutions and used at dilutions of 1:10000 and 1:1000, respectively. Monoclonal PrP specific mAb 8H4 (epitope 175–185, Abcam) was used at a dilution of 1:2000, while mAb 6H4 (epitope 140–152, Prionics) and mAb SAF70 (epitope 155–161, Bertin Pharma) were diluted 1:1000 (**Fig 1A**).

### Limited proteolysis of RT-QuIC reaction products

For each digestion experiment, PK (Merck Millipore) was diluted fresh on the day from a glycerol stock to working stock in cell culture grade water. PK working stock concentrations were calculated to digest RT-QuIC reaction products with different amounts of PK. The final digestion volume included 76 μL of sample and 4 μL of a PK working stock solution. Digestions were carried out at 37°C for 1 hour and stopped by placing the tubes on ice. PK activity was irreversibly inhibited by adding Pefabloc SC (Sigma-Aldrich) to 1.25 mM.

Products of PK digestion were methanol precipitated with 10 volumes of -20°C 99.9% pure methanol (Fisher Chemicals). Samples, with added methanol, were thoroughly vortexed and incubated overnight at -20°C. The following day samples were centrifuged for 35 minutes at 18200 x g (Eppendorf 5417R, rotor 06/09 HL128), the supernatant was discarded, and residual methanol evaporated by incubating the open tubes at 100°C in a Class II microbiological safety cabinet. Pellets were resuspended in 42 μL of 0.1%SDS/PBS, half of the volume was moved to a fresh tube and added to an equivalent volume of either LDS4X/2%βMA (reduced samples) or LDS4X with no βMA (not reduced samples).

### RT-QuIC data analysis

In experiments using 10%BH seeded RT-QuIC, in order to discriminate between fluorescence signal due to aggregation (signal) and the stochastic fluctuation of baseline fluorescence intrinsic to the RT-QuIC detection system (noise), a fluorescence threshold was calculated as equal to the average of the first fluorescence reading from each used well on a 96-well plate plus three standard deviations. Those reaction wells that produce three consecutive fluorescence readings equal or greater than threshold were deemed true positive signals and the time of the third fluorescence reading equal or above threshold was termed Time to Threshold (TtoT). The CSF seeded RT-QuIC reaction results were analysed with reference to the diagnostic criteria as described in [17].

## Results

### Seeding RT-QuIC with sCJD brain homogenate dilutions: BH-RT-QuIC

Currently, the molecular typing of the different sCJD subtypes is based on the genotyping of *PRNP* polymorphic codon-129 and the evaluation of the electrophoretic mobility and glycoform ratio of PrP^res^ as is assessed by WB using mAb [6,7]. Broadly, the discrimination of two different unglycosylated PrP^Sc^ PK digestion products (i.e. PrP^res^ Type 1 21 kDa and Type 2 19 kDa) suggests that PK exerts its activity on, at the very least, two different PrP^Sc^ conformations that are generated and conserved during the prion replication process *in vivo*.

Our hypothesis was that if seeds from sCJD subtypes specifically induced different misfolded conformations of the recombinant substrate in the RT-QuIC reaction products, then we could detect these alternate conformations by means of proteolytic treatment (PK digestion) and WB.

To study this, we initially generated RT-QuIC reaction products using sCJD 10% brain homogenates (10%BH) as RT-QuIC seeds, and then analysed them for their resistance to PK digestion. In addition, the protease resistant regions of PrP in the RT-QuIC reaction products were assessed by means of epitope mapping. The selected sCJD and non-CJD controls (i.e. 10%BHs) seeds were assayed in two RT-QuIC experiments. The amount of material used to seed individual RT-QuIC reactions with different 10%BHs was normalised to 100 fg of PrP^res^ by diluting the 10% BHs in PBS and an individual batch of recombinant substrate was used. At least six replicate reactions were performed for each seed in the same experiment.

**Fig 2:**
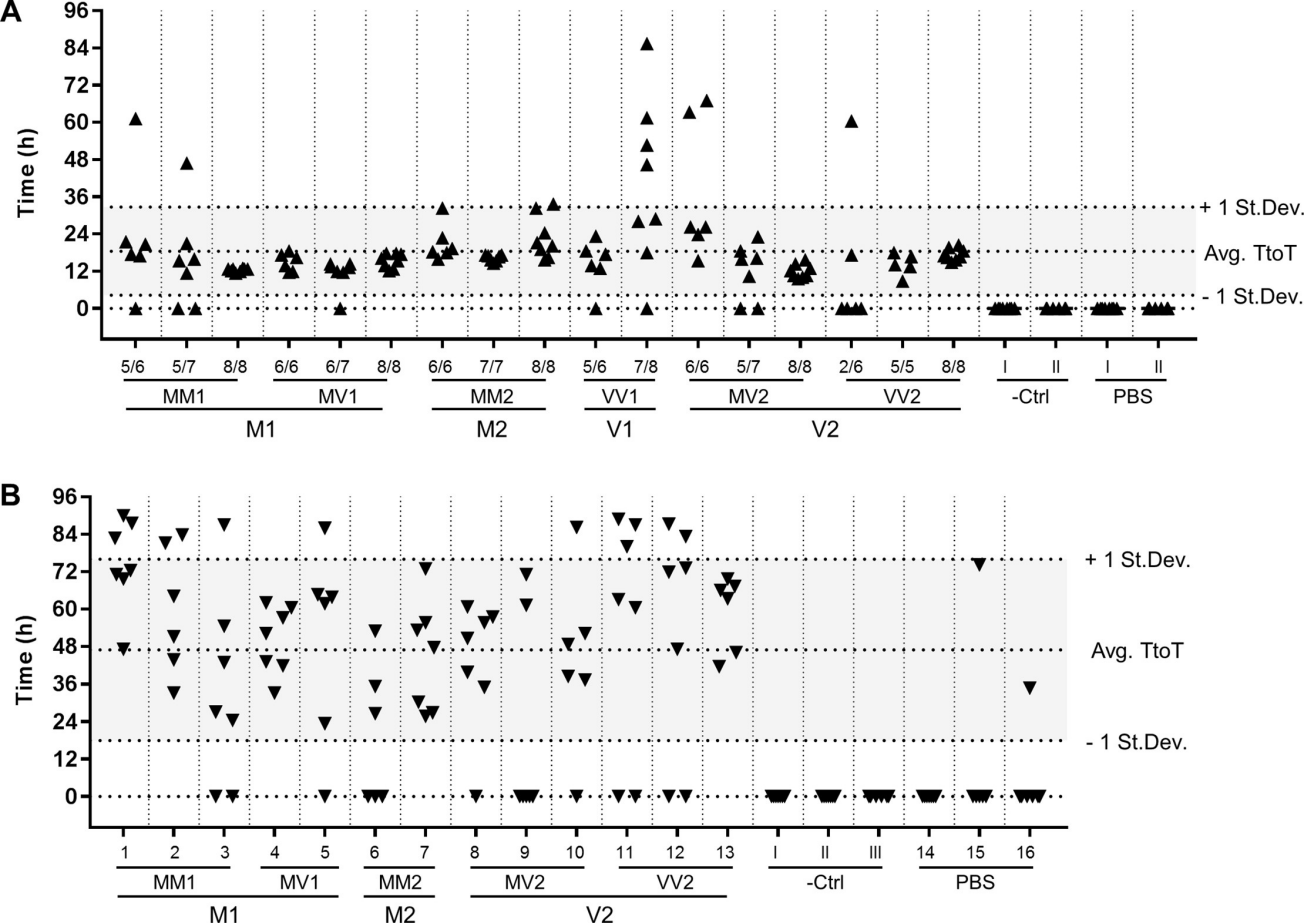
Graphical summary of the RT-QuIC reactions results. Each data point represents the time (in hours) at which an individual reaction well reached the threshold of fluorescence for positivity (Time to Threshold, TtoT). Technical replicates per individual seed are displayed in columns. Dashed lines mark the average TtoT +/- one standard deviation. (A) 10%BH seeded RT-QuIC (B) CSF seeded RT-QuIC. In both instances no subtype specific trend is observed.

While the spontaneous aggregation of the substrate was never observed in the negative controls or in the unseeded reactions, all sCJD seeded reactions showed aggregation-associated fluorescence for at least two replicates, and 89.2% of all the sCJD seeded wells showed fluorescence above threshold. No significant subtype specific trend, however, was observed (**Fig 2A**).

To investigate our hypothesis that the conformation(s) of the RT-QuIC reaction products are dependent on seed subtype, we sought to preserve the integrity of the misfolded conformation assumed by the recombinant substrate during RT-QuIC. With this aim, we collected the contents of individual reaction wells without using any detergent or denaturant. Densitometric analysis following WB with anti-PrP monoclonal antibody (mAb) 3F4 showed that it was not possible to recover the totality of the input substrate from individual reaction wells (i.e. 10 μg of FLHa-rPrP per well) and that the efficiency of collection was highly variable. By washing the wells with 0.1%SDS/PBS, and performing densitometric analysis on the collected washes, it was possible to show part of the input substrate was adherent to the well surface. Pooling replicated wells together reduced collection variability and, on average, 60 to 70% of the input recombinant substrate per group of replicates per seed was recovered.

**Fig 3:**
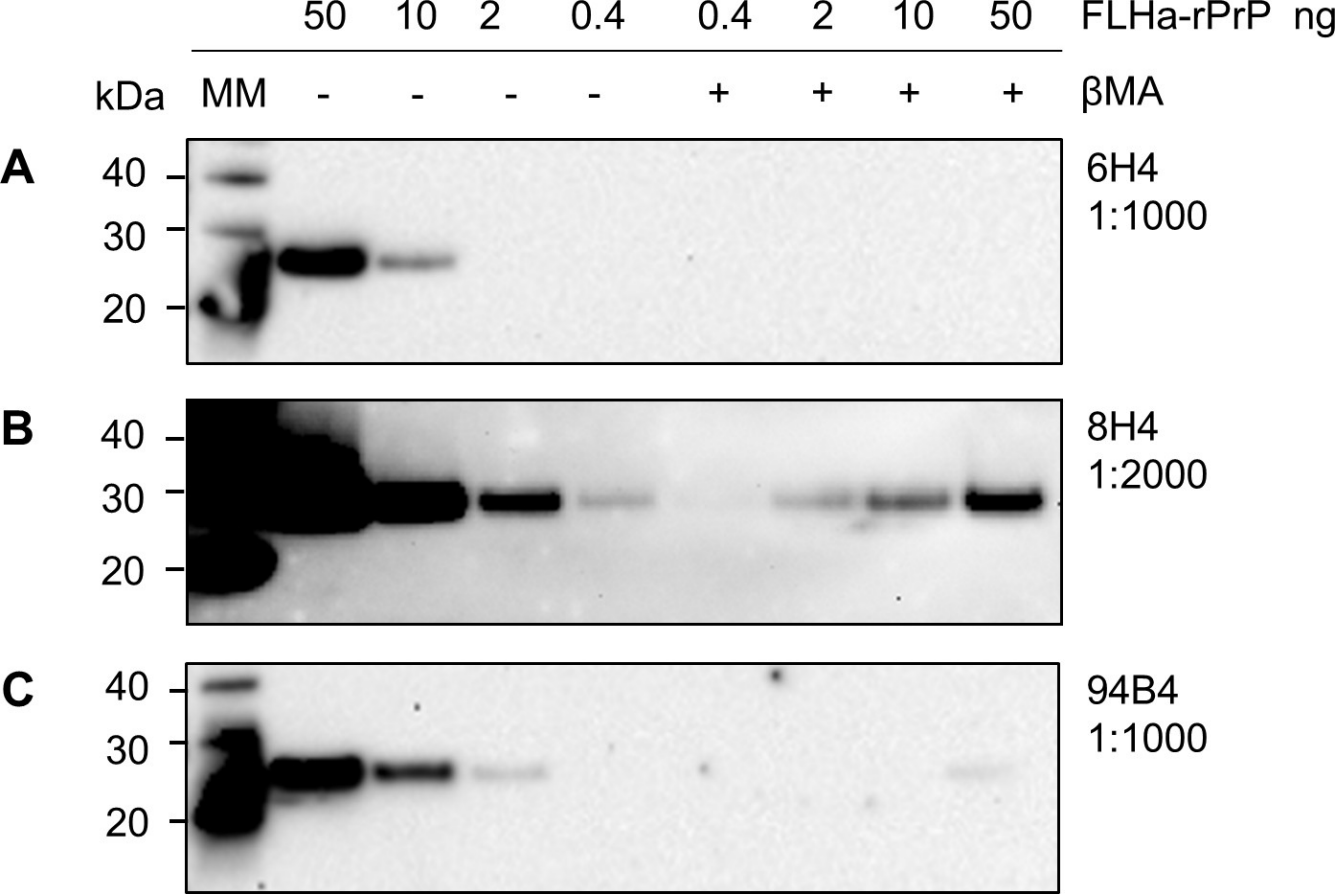
Sample reduction affects mAb reactivity. Dilutions of FLHa-rPrP of known concentration were boiled with (+) or without (-) 2% reducing agent β-mercaptoethanol. The dilution factor for each mAb is reported. All mAbs were diluted in TBST.

We then selected a panel of commercially available monoclonal antibodies to construct an epitope map of the RT-QuIC reaction products after proteolytic treatment with PK. The anti-PrP monoclonal antibodies (mAbs) sensitivity for detecting the RT-QuIC substrate (FLHa-rPrP) by WB was assessed. We found that the ability of mAbs 6H4, 8H4 and 94B4, (which are all directed towards PrP^C^ folded domains, **Fig 1A**), to detect of FLHa-rPrP was negatively affected by the presence of the reducing agent β-mercaptoethanol (**Fig 3**). Therefore, this component was excluded from the preparation of samples to be probed with these mAbs.

**Fig 4:**
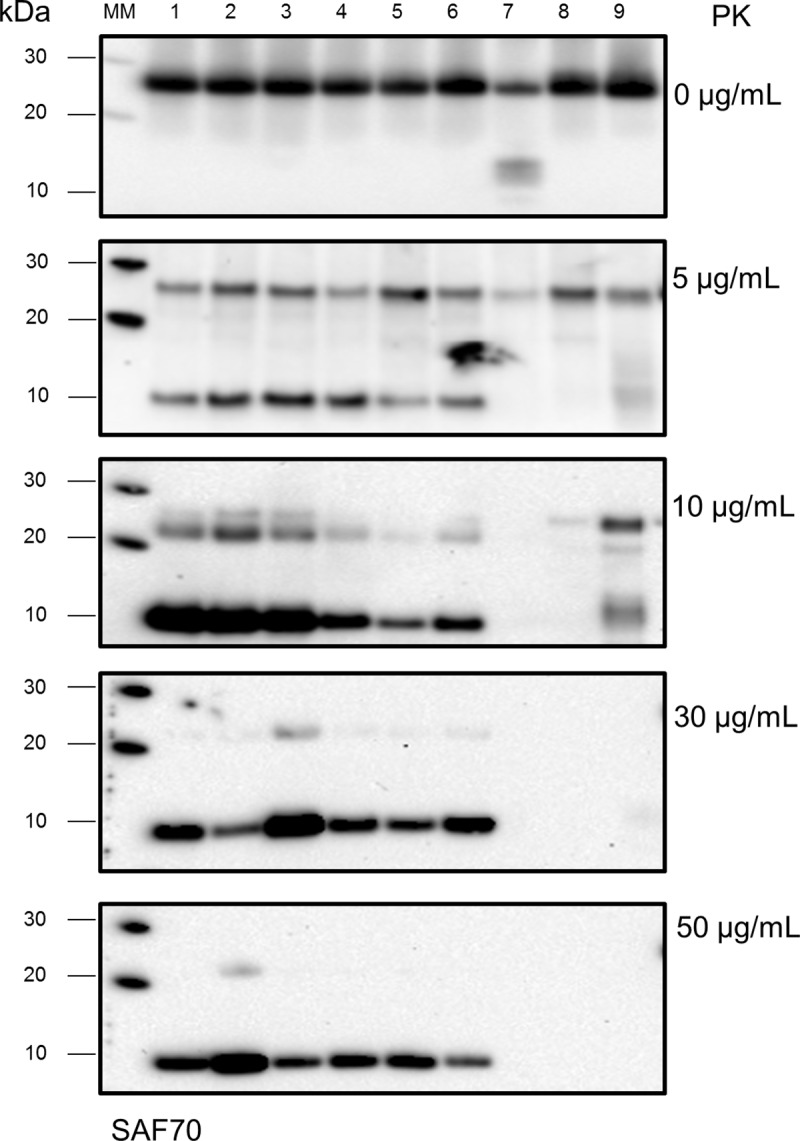
WB analysis with mAb SAF70 of RT-QuIC reaction products treated with increasing concentrations of PK. Lane 1–6: RT-QuIC reaction products from reactions seeded with sCJD subtypes MM1, MM2, MV1, MV2, VV1, VV2, respectively. Lane 7: products of RT-QuIC reactions seeded with non-CJD 10%BH. Lane 8: unseeded RT-QuIC reaction products. Lane 9: RT-QuIC untreated reaction mixture.

All the antibodies in our panel were able to detect the RT-QuIC reaction products prior to proteolytic treatment (**S1A Fig**). However, treating the RT-QuIC reaction products with PK at 10 μg/mL abolished or dramatically reduced the detection signal from all of the mAbs except SAF70 (epitope 155–161, **S1B Fig**), which also showed readily detectable resistant prion protein fragments after treatment with higher concentrations of PK (**Fig 4**).

RT-QuIC reaction products treated with PK 10 μg/mL and probed with mAb SAF70, showed a reduction in the signal intensity of the band corresponding to the full-length RT-QuIC product as well as the appearance of additional bands. In particular, a new band was detected at ~ 22 kDa as well as a prominent band at ~10 kDa (**Fig 4**).

It should be noted that the 22 kDa band was also detected in the RT-QuIC untreated Mix samples, whereas the 10 kDa band was found to be specific to sCJD seeded reactions, as it was not observed in unseeded or non-CJD seeded reactions or in the RT-QuIC untreated Mix.

**Fig 5:**
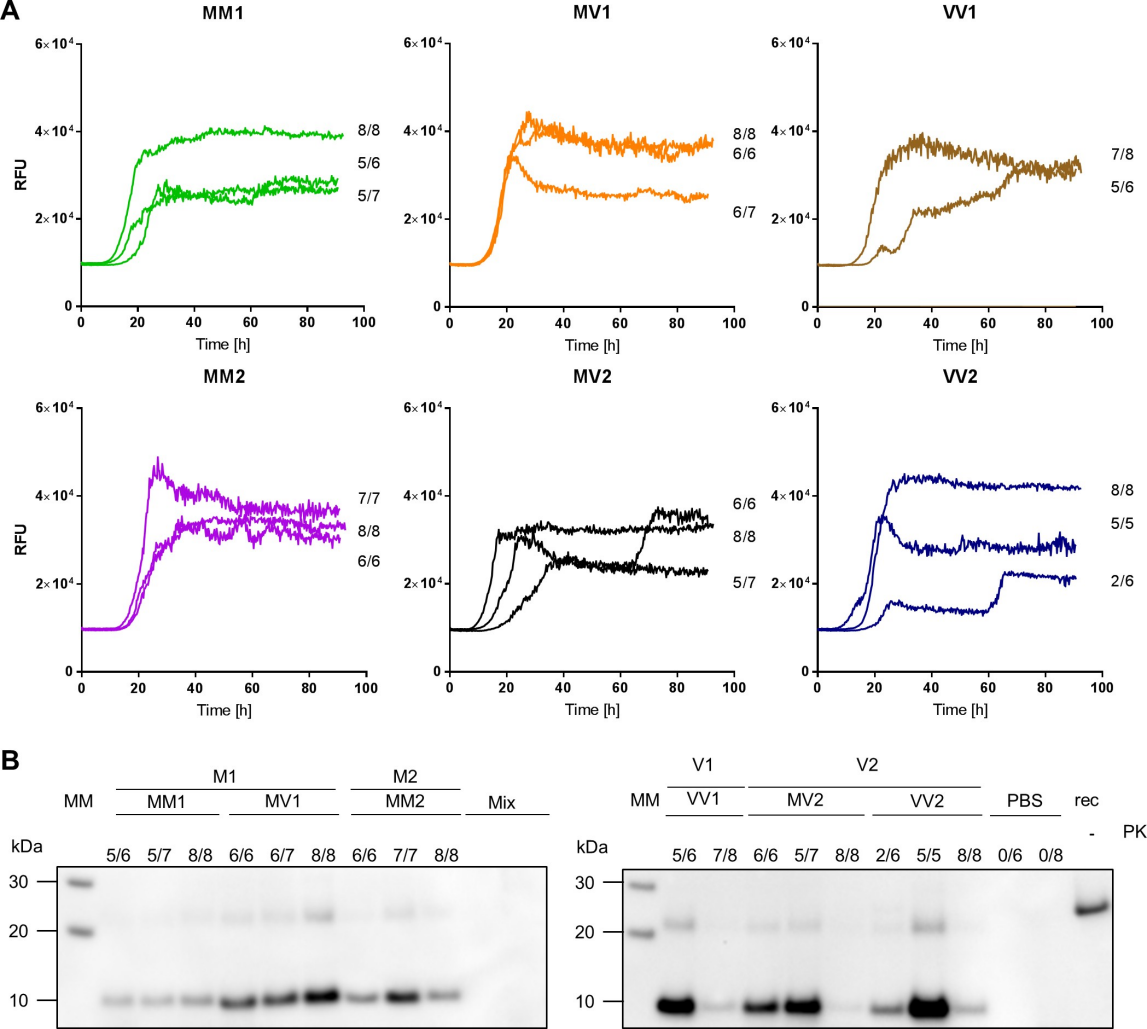
10%BH seeded RT-QuIC reaction kinetics and PK resistant RT-QuIC reaction products. A) Averaged RT-QuIC traces per individual seed are displayed along with the number of replicates that reached the fluorescence threshold for positivity. (B) RT-QuIC reaction products from reactions displayed in (A) were digested with PK (50 ug/mL) and probed by WB with mAb SAF70 1:1000. Samples are grouped according to sCJD strain of prions as defined in [8] and sub-grouped according to sCJD subtype as defined in [6]. Lane MM: Molecular weight marked; lane rec: 10 ng of FLHa-rPrP; Lane PBS: unseeded reactions; Lane Mix: RT-QuIC untreated reaction mixture.

In addition, the 10 kDa band was detectable by SAF70 even following treatment with 30 and 50 μg/mL PK, whereas the 22 kDa band was eliminated with these treatments. When treating samples with 30 or 50 μg/mL PK, occasionally, very faint bands were detected by mAbs other than SAF70 at a molecular weight corresponding to the intact FLHa-rPrP and more rarely at 22 kDa and 17 kDa (**S1C and S1D Fig**). SAF70, however, detected a similar if not identical banding pattern, characterised by a signal at 10 kDa, irrespective of the sCJD subtype used to seed the reaction (**Fig 5**).

### Seeding RT-QuIC with sCJD CSF samples: CSF-RT-QuIC

**Fig 6:**
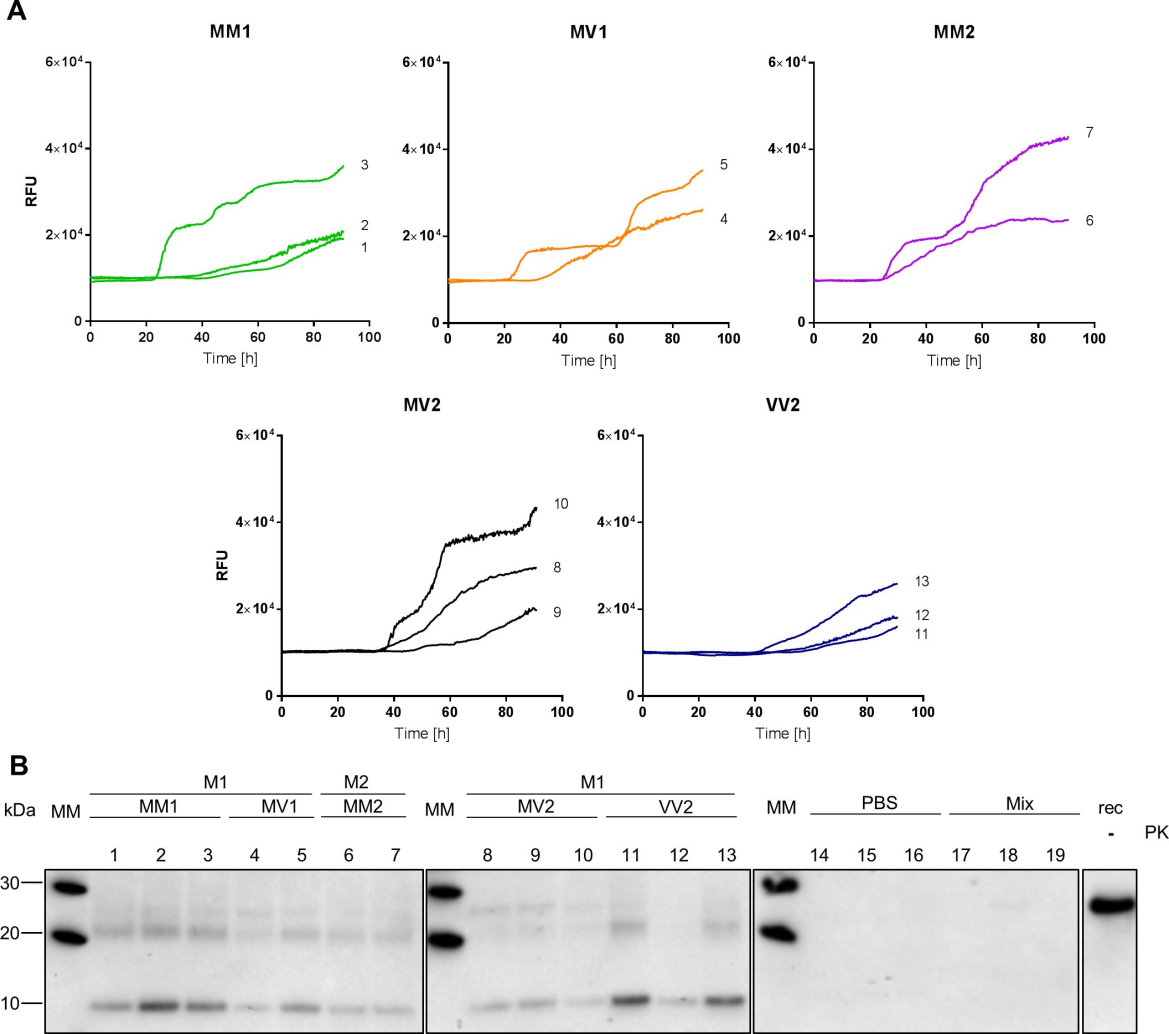
CSF seeded RT-QuIC reaction kinetic and PK resistant RT-QuIC reaction products. **(**A) Averaged RT-QuIC traces per individual seed are displayed (B) RT-QuIC reaction products from the reaction displayed in (A) were digested with PK 50 ug/mL and probed in WB with mAb SAF70 1:1000. Samples are grouped according to sCJD strain of prions as defined in (8) and sub-grouped according to sCJD subtype as defined in (6). Lane MM: Molecular weight marked; lane rec: 10 ng of FLHa-rPrP; Lane PBS: unseeded reactions; Lane Mix: RT-QuIC untreated reaction mixture.

Having established the WB methodology for the detection of RT-QuIC reaction products resulting from reactions seeded with 10%BH, we moved our study to the RT-QuIC reaction products resulting from seeding the reaction with CSF samples. Following a similar experimental design as with 10%BH, we assayed in three independent experiment the seeding activity of CSF samples from all the different sCJD subtypes except sCJD VV1. Upon testing by RT-QuIC, all the selected sCJD CSF samples satisfied the diagnostic criteria for positivity (17). Even though spontaneous aggregation of the substrate was observed in two out of 20 unseeded reaction wells, none of the negative controls showed aggregation-associated fluorescence. Treating CSF-RT-QuIC reaction products with 50 μg/mL PK and probing the digested material with mAb SAF70 in WB, resulted in a banding pattern characterised by two prominent signals at around 10 and 22 kDa irrespective of the sCJD subtype of the CSF sample used to seed the reaction. (**Fig 6**), consistent with the previous observation using sCJD brain homogenates.

## Discussion

The generation of PK resistant material from the prion seeded conversion of a recombinant substrate was first reported by Kocisko and co-workers [11] and has been exploited in different CFCs as a detectable sign of the prion seeded conversion of the recombinant substrates along with, or instead of, alterations in ThT fluorescence [32]. More recently, Sano and co-workers used two different rodent adapted prion strains and mouse rPrP as reaction substrates and showed that RT-QuIC reaction products retain some of the strain specific biochemical properties such as the conformational stability and β-sheet structure [27,29]. However, the same authors were unable to discriminate strain-specific structural differences by epitope mapping of PK digested RT-QuIC reaction products using the mAb ICSM35 (epitope 93–102) and the polyclonal PrP antiserum R20 (epitopes 218–231). These results were in line with previous findings obtained with sCJD seeds, showing that the conformation acquired by the seeded RT-QuIC reaction products confers PK-resistance to a C-terminal domain encompassing the R20 epitope [16]. In addition, the banding profile detected with R20 was specific to prion seeded RT-QuIC reaction products but independent of the rodent prion strain used as seed. Similar results have been obtained with R20 by McGuire and colleagues [23] on a limited number of PK-treated RT-QuIC reaction products generated with human 10%BH and CSF sCJD seeds and employing FLHa-rPrP as a reaction substrate. In both these studies only very mild PK treatments using 10 or 6 μg/mL PK were used, prior to western blotting.

The present study expands on these observations by systematically investigating the extent of PrP primary structure that is encompassed by the RT-QuIC PK resistant reaction products generated by sCJD seeds, by employing six commercially available monoclonal antibodies and harsher proteolytic conditions that are discriminatory for subtyping sCJD brain tissue itself.

The initial assessment of the sensitivity of the selected mAbs toward the RT-QuIC substrate FLHa-rPrP in WB, showed that the binding of mAbs 6H4, 8H4 and 94B4 to their respective epitopes was negatively affected when the reducing agent β-mercaptoethanol was added to the sample loading buffer (**Fig 3**). The reducing agent was added to reduce and thereby break the one disulphide bond in PrP (cysteine 179- cysteine 214, **Fig 1A**), favouring the linearisation of the protein structure by LDS and preventing the rPrP intermolecular dimerisation while in the denatured state. The fact that reducing conditions suppressed the binding of mAbs 6H4, 8H4 and 94B4 suggests that the correct presentation or availability of the epitopes recognised by these mAbs may depend on the local secondary structure of the protein, i.e. specific amino acid residues at the epitopes must be spatially arranged in a specific conformation. The disruption of the 6H4 epitope by reduction and alkylation has been reported previously [33]. Our data, however, show that reduction, even if acting distal to the 6H4 epitope, is sufficient to reduce or suppress 6H4 binding.

To our knowledge, this is the first report of the negative effect of sample reduction on 8H4 and 94B4 binding. However, in the latter case, the supplier (Central Veterinary Institute at Wageningen University, Netherlands, EU) states in the product information sheet that 94B4 epitope is “assumed to be conformation dependent” hence its binding may be susceptible to the conformational modifications determined by the reducing agent.

The cysteine at position 179 that forms one half of the disulphide bridge, is part of 8H4 epitope [34,35] and the latter is followed on the C-terminal side by the 94B4 epitope [36] (**Fig 1A**). Hence, the 8H4 and 94B4 epitopes are contiguous and map on one arm of the hairpin loop that may remain intact when the protein is denatured and the disulphide bond is oxidised. It is therefore conceivable that the disruption of the hairpin loop by reduction of the disulphide bridge may lead to a conformational rearrangement that in turn negatively affects the 8H4 and 94B4 epitopes.

In the absence of reducing conditions and when no PK treatment was used, all the antibodies in our panel detected both the FLHa-rPrP substrate prior to RT-QuIC and the FLHa-rPrP recovered from the plate following RT-QuIC (**S1A Fig**). The treatment of the RT-QuIC reaction products with 10 μg/mL PK, was sufficient to delete the signal detected by all the selected antibodies apart from SAF70 (**S1 Fig**). The fragment detected by SAF70 was specific to sCJD seeded RT-QuIC reactions and has an apparent molecular weight of around 10 kDa. The latter estimate was made on the basis of the migration distance on western blot, and is only therefore approximate. Nevertheless, the SAF70-detected band was observed irrespective of the sCJD subtype or whether brain or CSF was used to seed the reaction. Moreover, the fragment detected with SAF70 was unaffected by the harsher PK treatment used (i.e. 50 μg/mL PK, **Figs 5B and 6B**). Taken together these observations indicate the fragment detected by SAF70 represents the PK resistant conformational core generated during sCJD seeded RT-QuIC reactions.

The negative results obtained after PK digestion using mAbs that recognise more N-terminal epitopes, namely 12B2 and 3F4, indicate that these parts of FLHa-rPrP are digested by the mildest PK treatment used in this study. This observation is in line with previously published evidence regarding RT-QuIC reaction products generated with human and non-human prion seeds, indicating that in the conformation generated during RT-QuIC the C-terminal region of the recombinant substrate acquires PK resistance [16,22,29].

The apparent molecular weight of the fragment detected by SAF70 cannot be reconciled easily with the lack of detection by 6H4 and 8H4 because the region of hamster PrP between 6H4 and 8H4 epitopes has a much smaller molecular mass (~2.7 kDa) than the apparent molecular mass of 10 kDa (**Fig 1B**). Taking into consideration the susceptibility of 6H4 epitope to distal conformational changes that we have observed (**Fig 3A**), possibly the structural integrity of this epitope was affected by the proteolytic digestion of either or both the N-terminal and C-terminal domains, rather than a by direct effect of the PK.

It is also possible that the observed loss of 8H4 and 94B4 signals might also arise from a secondary conformational effect resulting from PK exerting its activity on distal domains so that the sequence encompassing these epitopes may contribute to the observed molecular weight of ~10 kDa. In particular, PK activity may determine an outcome similar to the one determined by the reducing agent (i.e. the disruption of the hairpin loop between α2 and α3), by exerting its activity on the hinge between the arms of the hairpin loop (**Fig 1A**). The digestion of the hinge may lead to a topological change in the local sequence that negatively affects 8H4 and 94B4 binding without destroying the peptide sequence encompassing their epitopes.

Our results show that the RT-QuIC reaction products, generated by seeding the reaction with either sCJD brain samples or sCJD CSF samples, are partially resistant to PK digestion. The PK digestion of the sCJD seeded RT-QuIC reaction products yields a fragment that is detected by mAb SAF70 at a molecular weight of ~10 kDa and the same banding profile is observed irrespective of the sCJD subtype used to seed the RT-QuIC reaction and irrespective to seed being a sCJD brain or CSF samples.

These results suggest that all the sCJD seeds have induced the same conformational change in the FLHa-rPrP as assessed by WB following PK digestion irrespective of PrP^res^ type or sCJD subtype of the seed and that the seeding mechanism is common to sCJD brain and CSF samples based on its effects on this particular commonly used substrate.

We cannot exclude the possibility that more complex and sensitive molecular methodologies, to the one used in this work, may be able to better characterise the products of the protein misfolding events taking place during RT-QuIC. These may eventually permit this technique to discriminate sCJD subtypes *in vita*. Nevertheless, our results do not support the hypothesis that there are distinctive misfolding pathways related to different sCJD subtypes that can be discerned using this commonly employed diagnostic RT-QuIC method. Therefore, whilst RT-QuIC has considerable diagnostic value, at present it has questionable prognostic value based on epitope mapping of reaction products.

## Supporting information

S1 Fig BH-RT-QuIC reaction products treated with increasing concentrations of PK and analysed by WB with panel of six commercially available mAs. Results from each individual mAb are displayed in columns, from left to right, 12B2 1:10000, 3F4 1:10000, 6H4 1:1000, SAF70 1:1000, 8H4 1:2000, 94B4 1:1000. This order mirrors the position on the FLHa-rPrP primary sequence of each mAbs epitope from the N- to the C-terminal. For each blot, Lanes (1–6): products of RT-QuIC reactions seeded with sCJD 10%BH subtypes MM1, MM2c, MV1, MV2, VV1, VV2, respectively. Lane (7): products of reaction seeded with non-CJD 10%BH. Lane 8: unseeded reaction products. Lane (9): RT-QuIC untreated reaction mixture. Row (A) RT-QuiC reaction products before proteolytic treatment, row (B) RT-QuIC reaction products treated with PK 10 μg/mL, row (C) PK 30 μg/mL, row (D) PK 50 μg/mL. Each row displays blots that were transferred on an individual PVDF membrane.
(TIF)
