# Supplementary figures and images for: Epitope mapping of the protease resistant products of RT-QuIC does not allow the discrimination of sCJD subtypes
Source: PLoS One. 2019 Jun 17;14(6):e0218509. doi: 10.1371/journal.pone.0218509 (PMC6576779; doi:10.1371/journal.pone.0218509)

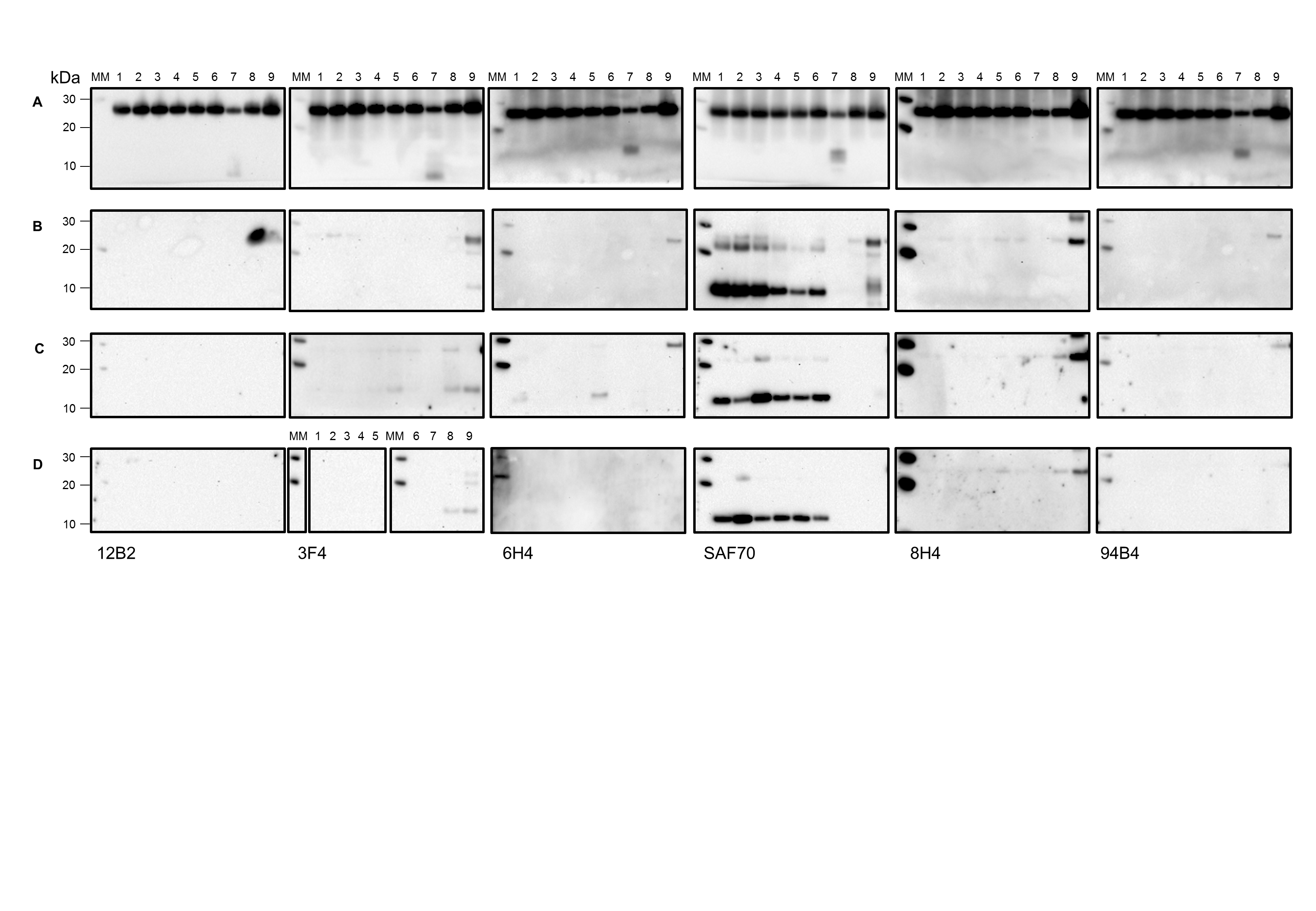

Supplement: S1 Fig — Results from each individual mAb are displayed in columns, from left to right, 12B2 1:10000, 3F4 1:10000, 6H4 1:1000, SAF70 1:1000, 8H4 1:2000, 94B4 1:1000. This order mirrors the position on the FLHa-rPrP primary sequence of each mAbs epitope from the N- to the C-terminal. For each blot, Lanes (1–6): products of RT-QuIC reactions seeded with sCJD 10%BH subtypes MM1, MM2c, MV1, MV2, VV1, VV2, respectively. Lane (7): products of reaction seeded with non-CJD 10%BH. Lane 8: unseeded reaction products. Lane (9): RT-QuIC untreated reaction mixture. Row (A) RT-QuiC reaction products before proteolytic treatment, row (B) RT-QuIC reaction products treated with PK 10 μg/mL, row (C) PK 30 μg/mL, row (D) PK 50 μg/mL. Each row displays blots that were transferred on an individual PVDF membrane. (TIF) [file pone.0218509.s001.tif]
